# Nanobodies mapped to cross-reactive and divergent epitopes on A(H7N9) influenza hemagglutinin using yeast display

**DOI:** 10.1038/s41598-021-82356-4

**Published:** 2021-02-04

**Authors:** Tiziano Gaiotto, Walter Ramage, Christina Ball, Paul Risley, George W. Carnell, Nigel Temperton, Othmar G. Engelhardt, Simon E. Hufton

**Affiliations:** 1 Biotherapeutics Division, National Institute for Biological Standards and Control, a Centre of the Medicines and Healthcare Products Regulatory Agency, Blanche Lane, South Mimms, Potters Bar, Herts EN6 3QG UK; 2 Division of Virology, National Institute for Biological Standards and Control, a Centre of the Medicines and Healthcare Products Regulatory Agency, Blanche Lane, South Mimms, Potters Bar, Herts EN6 3QG UK; 3 Infectious Diseases and Allergy Group, School of Pharmacy, University of Kent, Kent, ME4 4TB UK; 4 Present Address: Laboratory of Viral Zoonotics, Department of Veterinary Medicine, University of Cambridge, Cambridge, CB3 0ES UK

**Keywords:** Biological techniques, Biotechnology, Drug discovery, Immunology, Molecular biology

## Abstract

Influenza H7N9 virus continues to cause infections in humans and represents a significant pandemic risk. During the most recent 5th epidemic wave in 2016/17 two distinct lineages with increased human infections and wider geographical spread emerged. In preparation for any future adaptations, broadly reactive antibodies against H7N9 are required for surveillance, therapy and prophylaxis. In this study we have isolated a panel of nanobodies (Nbs) with broad reactivity across H7 influenza strains, including H7N9 strains between 2013 and 2017. We also describe Nbs capable of distinguishing between the most recent high and low pathogenicity Yangtze River Delta lineage H7N9 strains. Nanobodies were classified into 5 distinct groups based on their epitope footprint determined using yeast display and mutational scanning. The epitope footprint of Nbs capable of distinguishing high pathogenic (HP) A/Guangdong/17SF003/2016 from low pathogenic (LP) A/Hong Kong/125/2017 (H7N9) were correlated to natural sequence divergence in the head domain at lysine 164. Several Nbs binding to the head domain were capable of viral neutralisation. The potency of one nanobody NB7-14 could be increased over 1000-fold to 113 pM by linking two Nbs together. Nbs specific for distinct epitopes on H7N9 may be useful for surveillance or therapy in human or veterinary settings.

## Introduction

Influenza A virus (IAV) remains a persistent threat to public health resulting in 200,000–500,000 deaths worldwide annually which can be even higher in a pandemic emergency^1^. In the last century, four pandemics have led to many human deaths caused by three subtypes, H1N1 in 1918 and 2009, H2N2 in 1957 and H3N2 in 1968^2, 3^. In more recent years a number of avian influenza viruses, for example, H5N1 and H7N9, have crossed into the human population, but fortunately these zoonotic outbreaks have not yet become pandemics due to their low human-to-human transmissibility^4^. However, a more recent outbreak of H7N9 in China in 2016–2017 resulted in substantial geographical spread with the emergence of H7N9 strains carrying a polybasic cleavage site which can facilitate systemic spread beyond the lungs^5, 6^. As such, H7N9 may be considered a serious pandemic threat and there is a pressing need to prepare for robust global responses which can be rapidly implemented should this virus ever become highly transmissible within the human population. Vaccines remain the main prophylactic treatment option and in March 2017 the WHO recommended new candidate vaccine viruses be made from the Yangtze River Delta lineage of the low pathogenic A/Hong Kong/125/2017 (H7N9) [(LP)HK/2017(H7N9)] and the high pathogenic A/Guangdong/17SF003/2016 (H7N9) [(HP)GD/2016(H7N9)] viruses, which are currently being evaluated in clinical trials^7, 8^.

Although vaccines remain the mainstay of infection control for influenza, their timely implementation, poor immunogenicity in certain patient groups and limited worldwide production capacity remain a considerable technical challenge. Anti-viral drugs are available although resistant viral strains are emerging^9^. Passive transfer of serum from convalescent patients is an option which was deployed, with some success, in the 1918 pandemic^10^ and more recently to treat a severely ill H5N1 patient^11^. However, this approach cannot be implemented on a global scale due to limited supply, high risk of toxicity, uncertain dosing, and difficulties in administration. A more promising approach is passive immunotherapy with recombinant monoclonal antibodies with broad reactivity against H7N9 which can be manufactured with consistent quality, stockpiled, and delivered immediately in a pandemic emergency^12–14^.

As a typical member of the IAV family, H7N9 is classified through the major viral envelope glycoproteins, hemagglutinin (HA) and neuraminidase (NA). HA is a homo-trimeric molecule with each monomer consisting of two chains, HA1 and HA2 which fold into a structure comprising a highly variable globular head and a more conserved proximal stem domain^15^. HA mediates virus entry into host cells through the receptor binding site on the globular head interacting with sialic acid receptors on the cell surface. This in turn leads to virus internalisation, and then membrane fusion mediated by the HA stem region^15^. The pre-dominant host immune response is directed against the HA^16^ and this selective pressure drives the continuous antigenic changes in HA^17^. The high mutation rate and transmissibility means the discovery of new therapeutics is an active area of research^14^ and a number of broad spectrum human monoclonal antibodies (mAbs) specific for H7N9 have been described including CT149^18^, H7.167^19^, M826^20^, HNIgGD5^21^ HNIgGA6^22^ L4A-14^23^ and P52EO3^24^. These monoclonal antibodies have been identified from human donors after either natural IAV infection or H7 subunit vaccination^7, 19^ and unusually have limited somatic hypermutation. This suggests that they are the result of an early and immediate response to H7N9, likely biased by previous immune history of exposure to influenza^19, 23^. In addition, recent reports have suggested that an effective protective immune response to H7N9 infection is extremely rare in humans^23^. Alternative approaches to generating mAbs to H7N9 which have been extensively optimised*,* could be expected to yield molecules of higher affinity and to potentially different epitopes to those targeted by the human immune system.

Although HA is continually changing, there are certain parts of the HA that are highly conserved, principally because they are essential for the infection process. Two functionally conserved sites lie within the receptor binding site and the HA stem which are required for viral uptake and membrane fusion, respectively^15^. Several cross-reactive human antibodies have been isolated against the highly conserved HA stem^25, 26^ or the receptor binding site of the head domain^27^. In several cases the route to cross-reactivity is achieved through using only their heavy chain for binding or through using a smaller binding footprint^25–27^. Conceptually a much smaller antibody epitope footprint may give a variable HA antigen less opportunities to escape binding and in effect there will need to be a mutational ‘bullseye’ for the virus to evade binding. Guided by these observations we have used nanobodies (Nbs) from alpacas as a potential route to cross-reactive antibodies^28–30^ as they are naturally devoid of a paired light chain and have a propensity to bind small pockets on protein surfaces^31^. In addition, given that camelid species are not thought to represent a zoonotic reservoir for influenza A, they may not be subject to the same immune constraints as human donors with prior exposure to IAV^32^. The unique properties of Nbs which also include small size, simple engineering into multi-domain antibodies and high stability are being exploited for a wide range of applications in biotechnology including diagnostics and immunotherapy^31, 33^, with the first nanobody, Caplicizumab, having been approved for the treatment of a blood clotting disorder in August 2018^34^.

In this study we have isolated Nbs with both cross-reactive and strain specific binding to the prototype low pathogenicity (LP) and high pathogenicity (HP) A(H7N9) strains which have emerged in the most recent 5th epidemic wave. We correlate their specificity and functional activity with their epitopes which have been mapped using yeast display and mutational scanning. This technique allows high throughput epitope mapping and identifies mutations that directly interfere with antibody binding. Their potential as tools for surveillance or emergency therapeutics in both human and veterinary applications is discussed.

## Results

### Isolation and characterisation of nanobodies specific for H7 hemagglutinin (HA)

**Figure 1:**
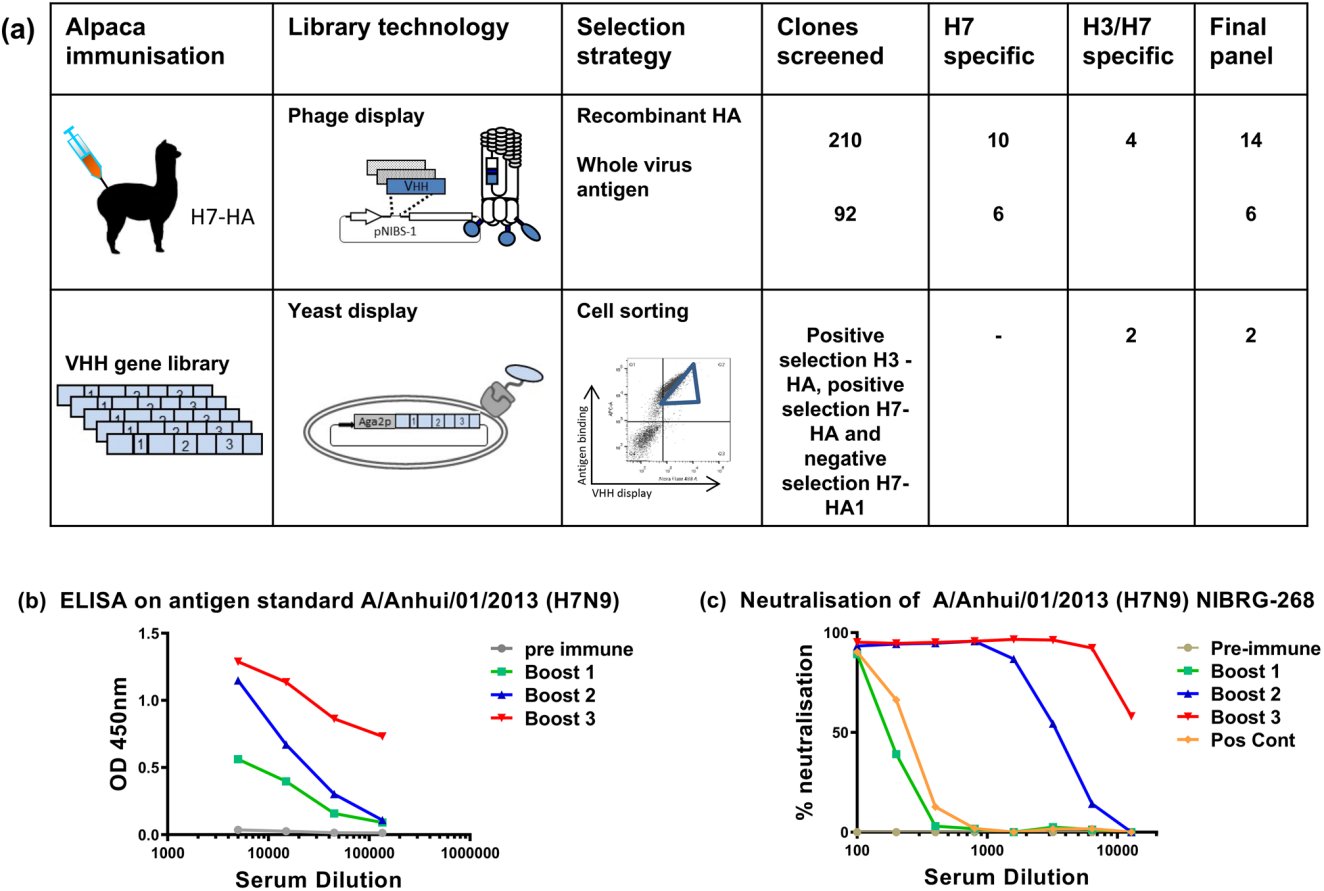
Isolation of H7-HA specific nanobodies. (**a**) The number of clones screened and the number of H7 or H3/H7 specific Nbs identified are shown from either phage display or yeast display library selection (Fig S1). (**b**) Serological immune response in immunised alpaca to A/Anhui/01/2013 (H7N9). Samples were taken pre-immunisation and after each of three boosts. (**c**) Serological neutralising immune response in immunised alpacas using micro-neutralisation assays of A/Anhui/01/2013 (H7N9) NIBRG-268. A positive control sheep serum specific for the reverse genetics virus was used as a positive control.

A juvenile male alpaca was immunised with purified recombinant H7-HA (Fig. 1a) from the human H7 strain A(H7N7) HA (A/Netherlands/219/2003) and an antigen specific serological immune response was seen in both ELISA and viral micro-neutralisation assays (Fig. 1b,c). After a third booster injection a phage displayed nanobody library of 9.6 × 10^7^ was constructed using purified peripheral blood mononuclear cells. Two phage display library selection strategies were used, (i) sequential rounds of selection on the same antigen to yield all Nbs specific for H7-HA (H7N9 or H7N7) or (ii) alternating selections between H7-HA and H3-HA to bias towards cross-subtype reactive Nbs. Primary screening involved picking random clones and testing for binding to recombinant HA and whole virus inactivated antigen reagents of subtypes H3 and H7 sub-types as non-purified Nbs. Clones that were positive in ELISA were then sequenced and grouped into clonally related families based on their VHH-CDR3 sequences (Table 1). Nbs with homologous VHH-CDR3 sequences of identical length were taken as belonging to the same clonal family and predicted to recognise the same or closely related epitopes. Several families of nanobodies containing homologous VHH-CDR3 sequences were identified which were likely derived from the same B cell lineage (Table 1). Nbs were purified and screened in ELISA on a panel of whole virus H7 antigen preparations to assess the breadth of cross-reactivity across both avian and human H7 strains from 2000 up to the most recent 5th epidemic wave of H7N9 in 2016–17. Most Nbs were capable of homo-subtypic cross-reactivity against influenza H7 strains, including H7N9 strains from 2013 to 2017 such as (HP) A/Guangdong/17SF003/2016 (H7N9) [(HP)GD/2016] and (LP) A/Hong Kong/125/2016 (H7N9) [(LP)HK/2017] Yangtze River Delta Lineage strains. We also identified a subgroup of Nbs (NB7-03, NB7-10 and NB7-13) which had lost binding to the most recent (HP)GD/2016 H7N9 strain. Nanobodies NB37X-01 – 04 were a distinct group with hetero-subtypic cross-reactive binding which extended to include the group 2 strain A/Texas/50/2012 (H3N2).

**Table 1:** Nanobody sequence, specificity and neutralisation activity.

Nb	Sequence	Binding specificity (inactivated whole virus antigen)						Neutralisation	
	VHH CDR3 sequence (family)	TX/2012 (H3N2)	M/N/2000 (H7N3)	NY/2003 (H7N2)	AN/2013 (H7N9)	GD/2016 (H7N9)	HK/2017 (H7N9)	TX 2012 (H3N2)	SH 2013 (H7N9)
NB7-01	ALARSVDRLTYDY (1)	-	2.83	2.53	2.26	2.59	1.94	-	++++
NB7-02	HASPRDVLVDYDY (2)	-	1.98	2.44	1.63	2.48	0.26	-	++
NB7-03	AADFLTLQDMCVFSPITRYHD (3)	-	2.04	-	1.30	-	0.52	-	+++
NB7-04	AASPIPDLRDYDY (4)	-	0.84	1.62	0.71	3.37	0.31	-	+
NB7-05	NVWIQFHNY	-	0.17	-	0.19	-	0.38	-	-
NB7-07	AFGATLYGVRRDEYAY	-	1.99	1.96	1.46	2.73	0.48	-	+++
NB7-08	AADRYSGPYDLGTISPRQDHYDV	-	0.91	1.52	0.91	2.65	2.05	-	-
NB7-09	ALARSVDRLEYDW (1)	-	2.86	2.82	2.37	3.30	2.60	-	++++ +
NB7-10	AADDLTLQDMCVMSPISRYHY (3)	-	1.64	-	1.20	-	2.30	-	+++
NB7-11	AVARSVDRLEYDY (1)	-	1.03	1.23	1.24	3.12	2.53	-	++++
NB7-12	HASPRDVLVDYD (2)	-	1.73	2.57	1.78	3.28	2.47	-	++++
NB7-13	AADFLTLQDMCVLSPITRYRS (3)	-	1.14	-	0.72	-	2.37	-	+++
NB7-14	ALARSVDRLTYDY (1)	-	2.67	2.75	2.08	3.00	2.57	-	+++++
NB7-15	AAGRTYCGLRGYEYDY	-	2.23	0.20	1.92	2.97	2.56	-	++++
NB7-16	AASPIPTLRDYDY (4)	-	1.44	1.71	1.08	2.86	2.42	-	+++
NB7-17	AASPRRLMCVPAEFDLYY	-	2.44	2.57	1.97	2.39	2.07	-	++++
NB37X-01	AQERERIGTTIRYY	0.36	0.13	0.22	0.48	0.38	2.14	-	-
NB37X-02	AAPKTRYLAPRAMESEFDY (5)	2.72	-	0.12	-	0.26	2.48	-	-
NB37X-03	AAPKTRYLAPRMTESDYDY (5)	0.12	-	-	-	0.34	-	-	-
NB37X-04	NALCYGCTPSSY	0.30	0.28	-	0.14	0.49	2.83	-	-
NB37X-05	AANPGTVVGRTRLSPRLRVPDEYDS	0.40	0.14	0.17	0.26	0.31	2.32	-	-
NB37X-06	AADRVDYPGGGATCRTSSAAYDH	0.26	-	0.20	0.20	0.36	2.55	-	-

VHH-CDR3 sequences and family grouping of related sequences. ELISA is given as the average OD450nm reading of two wells coated with whole virus antigen and Nbs added at a single concentration of 5 µg/ml. – indicates OD450 < 0.1 and represents no binding. Inactivated whole virus antigens used TX/2012(H3N2) [A/Texas/50/2012 (H3N2)], M/N/2000(H7N3) [A/Mallard/Netherlands/12/2000], NY/2003 (H7N2) [A/New York/107/2003], AN/2013 (H7N9) [A/Anhui/01/2013], GD/2016 (H7N9) [A/Guangdong/17SF003/2016], HK/2017 (H7N9) [A/HongKong/125/2017]. Pseudotype based micro-neutralisation assays using A/Texas/50/2012 (H3) and A/Shanghai/02/2013 (H7) HA bearing pseudoviruses. += 500-3000 nM, ++ < 500 nM, +++< 100 nM, ++++  < 50 nM, +++++< 10 nM, - no neutralisation.

We used yeast display to identify further hetero-subtypic H3/H7 cross-reactive Nbs that may have been missed using conventional phage display library screening. This technology has advantages over phage display as highly controlled selection strategies are possible where each individual yeast cell can be quantitatively selected for multiple parameters using flow cytometric cell sorting^28, 35, 36^. The VHH genes from the immunised alpaca were cloned into a yeast display vector (Fig. 1a) and the resulting library sorted for H7-HA binding and then subsequently with H3-HA (Fig. S1). By co-labelling with an anti-SV5 epitope tag, antibody antigen binding could be normalised to display level. Cross-reactive Nbs were predicted to bind to more conserved epitopes in the HA stem region^28^, so we performed a third round of ‘negative’ sorting using purified recombinant HA1 head domain, selecting cells displaying Nbs that do not bind the head domain (Fig. S1). Sequence analysis of randomly picked yeast clones from this epitope guided yeast library selection strategy gave exclusively H3/H7 cross-reactive nanobodies demonstrating the precision possible using cell sorting of yeast displayed libraries. In addition, two new Nbs (NB37X-05 and NB37X-06) with unique CDR3 sequence were identified which were not recovered using phage display.

**Figure 2:**
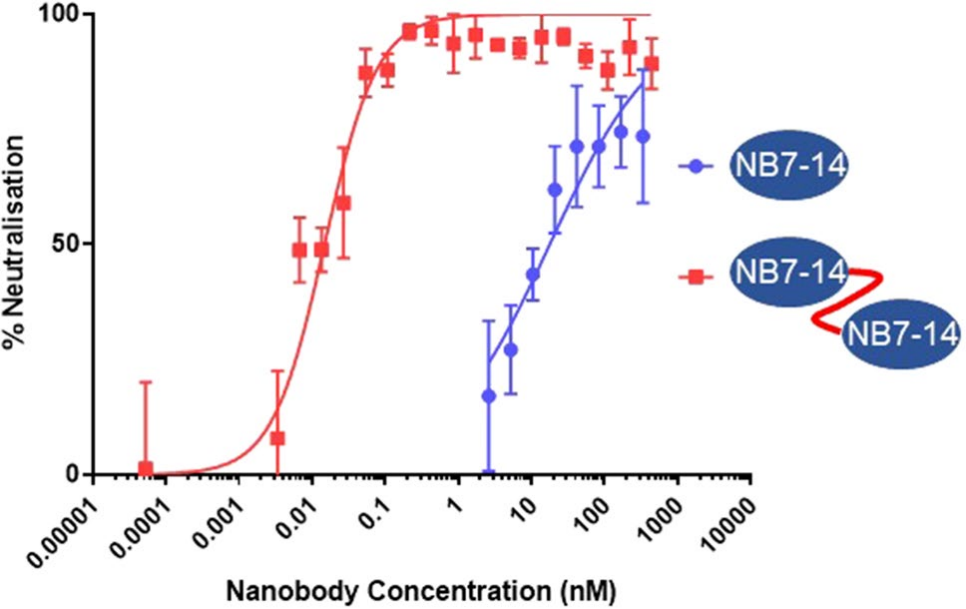
Comparison of neutralisation of H7N9 by monovalent and bivalent NB7-14. Pseudotype micro-neutralisation assay performed using influenza A/Shanghai/02/2013 (H7) HA bearing pseudovirus and mono- and bivalent nanobody NB7-14. Percentage neutralisation is plotted against molar concentration of NB7-14 in two different formats (nM). IC50 for monovalent NB7-14 (17 nM) and for bivalent NB7-14 (130 pM). Neutralisation of A/Texas/50/2012 (H3N2) was negative (data not shown).

The final panel of 22 Nbs was then screened for neutralisation activity using pseudotyped viruses displaying the HA of A/Shanghai/02/2013 (H7N9) (Table 1). Several Nbs were neutralising with the most potent group being NB7-01, NB7-09, NB7-11 and NB7-14 which all belonged to the same clonal lineage with homologous VHH-CDR3 sequences of identical length. One of the advantages of Nbs over conventional mAbs is that they can be easily reformatted into multi-domain antibodies as all antigen binding activity is confined to a single domain. Linking Nbs together has been shown to significantly increase their potency through avidity^29, 37^ and we investigated whether similar increases in potency were possible with the lead nanobody NB7-14. Bivalent versions of NB7-14 were produced by fusing two identical binding domains separated by a thirty amino acid glycine linker and saw an increase in potency of more than 3 orders of magnitude to an IC50 (half maximal inhibitory concentration) of 113 pM (Fig. 2). Previous studies have shown that a bivalent nanobody specific for the head domain of H5N1 had substantially increased potency compared to the monovalent counterpart in both in vitro neutralisation assays and a mouse challenge model^38^. Our findings agree with these studies and suggest bivalent NB7-14 has improved potency through enhanced avidity and cross-linking of HA through either inter- or intra- molecular interactions.

### Epitope mapping of H7-HA specific nanobodies

To group Nbs as either head or stem specific we evaluated binding to purified recombinant HA1 head domain and full length HA0 protein using surface plasmon resonance and single cycle kinetics^39^. H7-specific Nbs (NB7-01 to 17) showed high affinity binding to both the HA1 domain and full length HA0 (Table 2) indicating that their epitopes lie in the globular head domain. Several Nbs had affinities less than 1 nM as a monovalent binding affinity constant (Fig. S2) and binding affinity was retained across all H7 strains tested including the most recent A/HongKong/125/2017 (H7N9). The hetero-subtypic cross-reactive nanobodies NB37X-01 to 06 did not show any binding to the head domain, however, they showed some degree of binding to full length HA0, but the data could not be fitted using a 1:1 model. This suggests that this group of H3/H7 cross-reactive nanobodies bind to epitopes outside of the head domain and likely to epitopes within the HA stem region.

**Table 2:** Characterisation of nanobody binding affinity and head/stem specificity.

Nanobody	Affinity (KD)			
	A/Brisbane/10/2007 (H3N2) HA0^2^	A/Chick/Netherlands/2003 (H7N7) HA0^2^	A/Shanghai/01/ 2013 (H7N9) HA0^2^	A/HongKong/125/ 2017 (H7N9) HA1^1^
NB7-01	-	< 0.1 nM	4.80 nM	0.83 nM
NB7-02	-	6.19 nM	17.3 nM	6.24 nM
NB7-03	-	24.80 nM	35.70 nM	27.45 nM
NB7-04	-	8.74 nM	26.9 nM	10.79 nM
NB7-05	-	140 nM	6.96 nM	> 1 µM
NB7-07	-	0.81 nM	4.31 nM	0.78 nM
NB7-08	-	1.26 nM	2.22 nM	15.48 nM
NB7-09	-	< 0.1 nM	0.38 nM	0.93 nM
NB7-10	-	24.0 nM	21.6 nM	46.24 nM
NB7-11	-	4.60 nM	58.8 nM	18.74 nM
NB7-12	-	1.05 nM	1.50 nM	3.72 nM
NB7-13	-	> 1 µm	–	> 1 µm
NB7-14	-	< 0.1 nM	2.36 nM	0.41 nM
NB7-15	-	0.42 nM	1.24 nM	0.77 nM
NB7-16	-	1.63 nM	9.98 nM	4.95 nM
NB7-17	-	1.43 nM	1.30 nM	2.40 nM
NB37X-01	#	-	#	-
NB37X-02	#	-	#	-
NB37X-03	#	-	#	-
NB37X-04	#	-	#	-
NB37X-05	#	-	#	-
NB37X-06	#	-	#	-

^1^ HA1 is purified hemagglutinin head domain.

^2^ HA0 is purified full length hemagglutinin.

^#^ binding seen but could not be fitted using 1:1 fitting model.

- no binding could be seen.

For precise epitope localisation we used yeast surface display coupled with mutational scanning which we have described previously^28^. The HA0 precursor gene of hemagglutinin A/Netherlands/219/2003 (H7N7) was sub-cloned into a yeast display vector in frame with an SV5 epitope tag. The use of a C-terminal epitope tag allows the detection and sorting of correctly folded HA displayed on the yeast surface independently of Nb binding using flow cytometry (Fig. S3). All Nbs showed binding to yeast cells displaying H7-HA0 suggesting that the epitopes could be located.

**Figure 3:**
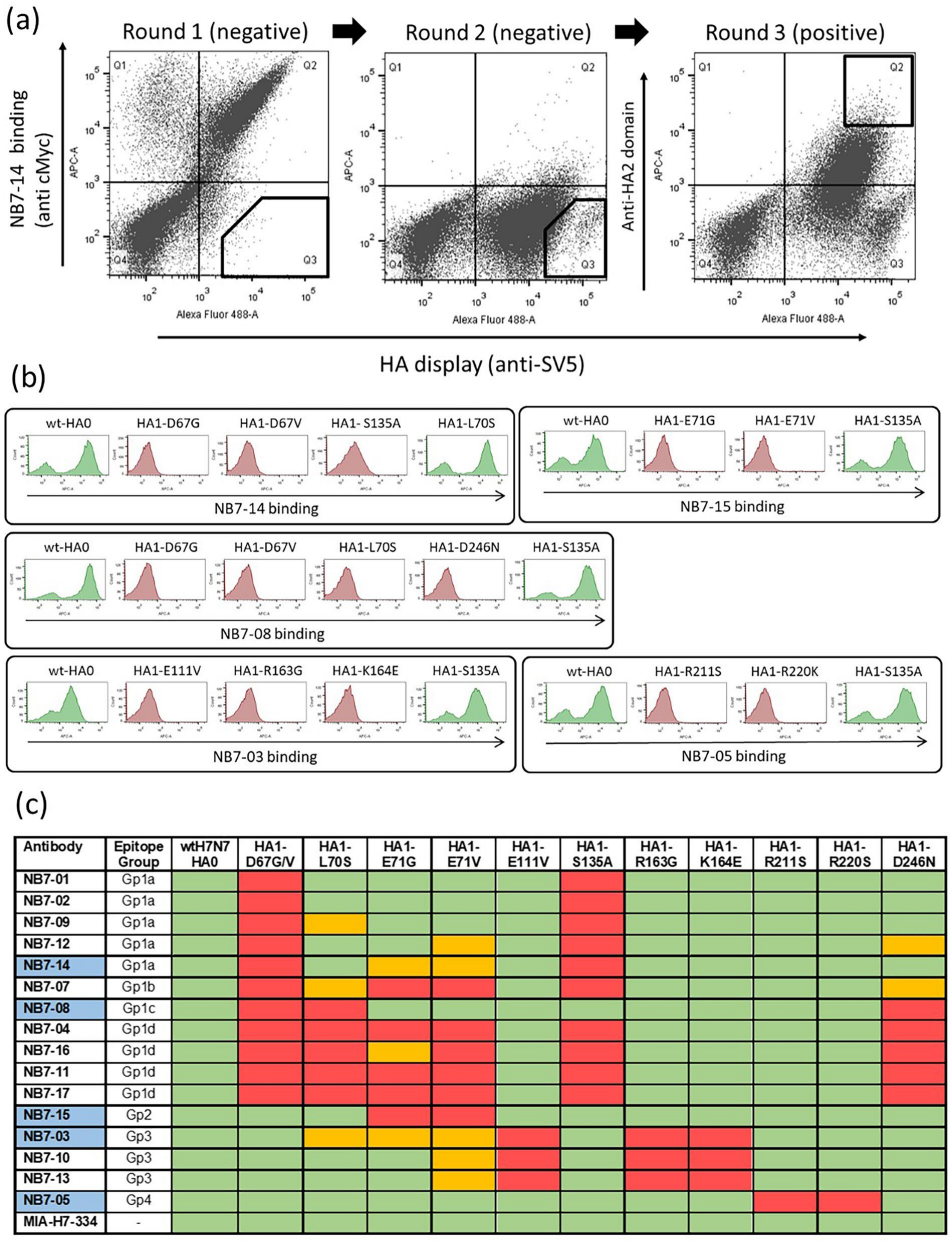
Epitope footprints of H7-HA specific nanobodies. (**a**) Example selection campaign for NB7-14 showing two rounds of ‘negative’ selection followed by a third round of ‘positive’ selection. (**b**) Flow cytometry histograms (FlowJo 10.4 software) showing Nb binding to yeast displayed wild-type H7-HA0 (A/Netherlands/219/2003) and binding of NB7-14, NB7-08, NB7-15, NB7-03, NB7-05 to yeast displayed HA carrying the mutations as indicated. (**c**) Binding activity to a panel of yeast displayed H7-HA0 mutants with residue numbering relative to HA1 domain. Each Nb is grouped by binding profile. Blue cells indicate the specific Nb used for HA library selections. Commercial H7-HA specific antibody MIA-H7-334 is included as positive control and retains binding to all mutants. Relative Nb binding to each yeast displayed HA mutant was calculated by dividing the MFI (mean fluorescence intensity) of Nb-mutant HA pair with the value for wild type H7-HA incubation and the resulting ratio normalised to percentage values. Binding was categorized as follows, ≤ 15% no binding (red), between 15 and 40% intermediate binding (orange) and ≥ 40% positive binding (green).

Subsequently, a library of H7-HA0 mutants was generated by error-prone PCR using low-error rate mutagenesis. Initially the library was selected by incubating with NB7-14 and NB7-08 separately, and mutants which completely lost binding were recovered through two sequential rounds of ‘negative’ flow cytometric cell sorting. As allosteric mutations distant from the actual physical binding footprint could, in principal, lead to loss of binding, we performed a third round of ‘positive’ sorting with a non-competing antibody targeting the stem domain (Fig. 3a). This selection step focuses the recovery of mutations which lie within the physical epitope footprint of NB7-08 and NB7-14 and biases against HA mutations that may have an indirect effect on binding through structural disruption distant from the site of physical interaction. Following selection, random clones (*n* = *30*) were picked, sequenced and aligned to wild-type HA0 to identify HA mutations which were enriched compared to the unselected library. These selectively enriched mutations were predicted to form part of the physical epitope footprint and were chosen for further testing. Clones with mutations introducing/replacing cysteine or proline residues were discarded as they were predicted to have indirect or pleiotropic effects on binding^28^. Only HA mutations which were shown to interfere with Nb binding without affecting HA display level were classified as epitope residues. Selection for loss of binding to NB7-08 identified mutations D67G, D67V, L70S and D246N located in the HA1 domain, (Fig. S4). D67 was mutated to both glycine and valine, reflecting its importance within the epitope of NB7-08. For NB7-14, HA-D67G was also selected, however HA-S135A mutation was also enriched (Fig. 3b,c) reflecting NB7-14 has a distinct but overlapping epitope footprint with NB7-08.

Each individual HA mutation was subsequently tested for binding to the complete panel of H7-HA specific Nbs (Fig. 3b,c) to allow grouping Nbs with overlapping epitopes. Although 11/16 Nbs lost binding due to a mutation at D67 and could be classed as broadly belonging to a single epitope group (Gp1) it was clear that epitope groups could be further de-lineated based on the individual mutations capable of disrupting binding.

The remaining Nbs (NB7-03, NB7-05, NB7-10, NB7-13 and NB7-15) that were not affected by the D67 mutations required further HA library selections to identify their respective epitopes. In selections with NB7-15, two separate mutations at residue 71 (E71G and E71V) were selected and placed NB7-15 in a separate epitope group (Gp2). Selections with NB7-03 gave mutations E111V, R163G and K164E, again placing it in a further separate epitope group, Gp3 (Fig. 3b,c). Residue E111 was identified as particularly important as it was recovered multiple times mutated to different residues E111(G/D/K/R). Since binding of NB7-05 was not affected by any of the previously selected HA mutations, additional selections were done and R211S and R220S were shown to specifically disrupt its binding, placing it in epitope group 4 (Gp4) (Fig. 3b,c).

All selected mutations were finally tested against the whole panel of Nbs which allowed the Nbs to be placed into 4 distinct epitope binding groups (Gp1, Gp2, Gp3, Gp4) with Gp 1 being divided into subgroups (Gp1a, Gp1b, Gp1c, Gp1d )(Fig. 3b,c) based on different combinations of mutations with D67. All mutations were mapped onto the structure of H7-HA and could be seen to be surface exposed residues, which suggests a direct involvement in Nb binding rather than a more general structural disruption (Fig. 5). Nbs belonging to the same epitope grouping in several cases had unique VHH CDR3 sequences reflecting their unique paratopes. This is an important consideration as, although they may have overlapping epitopes, they might present a different genetic barrier to a constantly changing influenza HA antigen.

**Figure 4:**
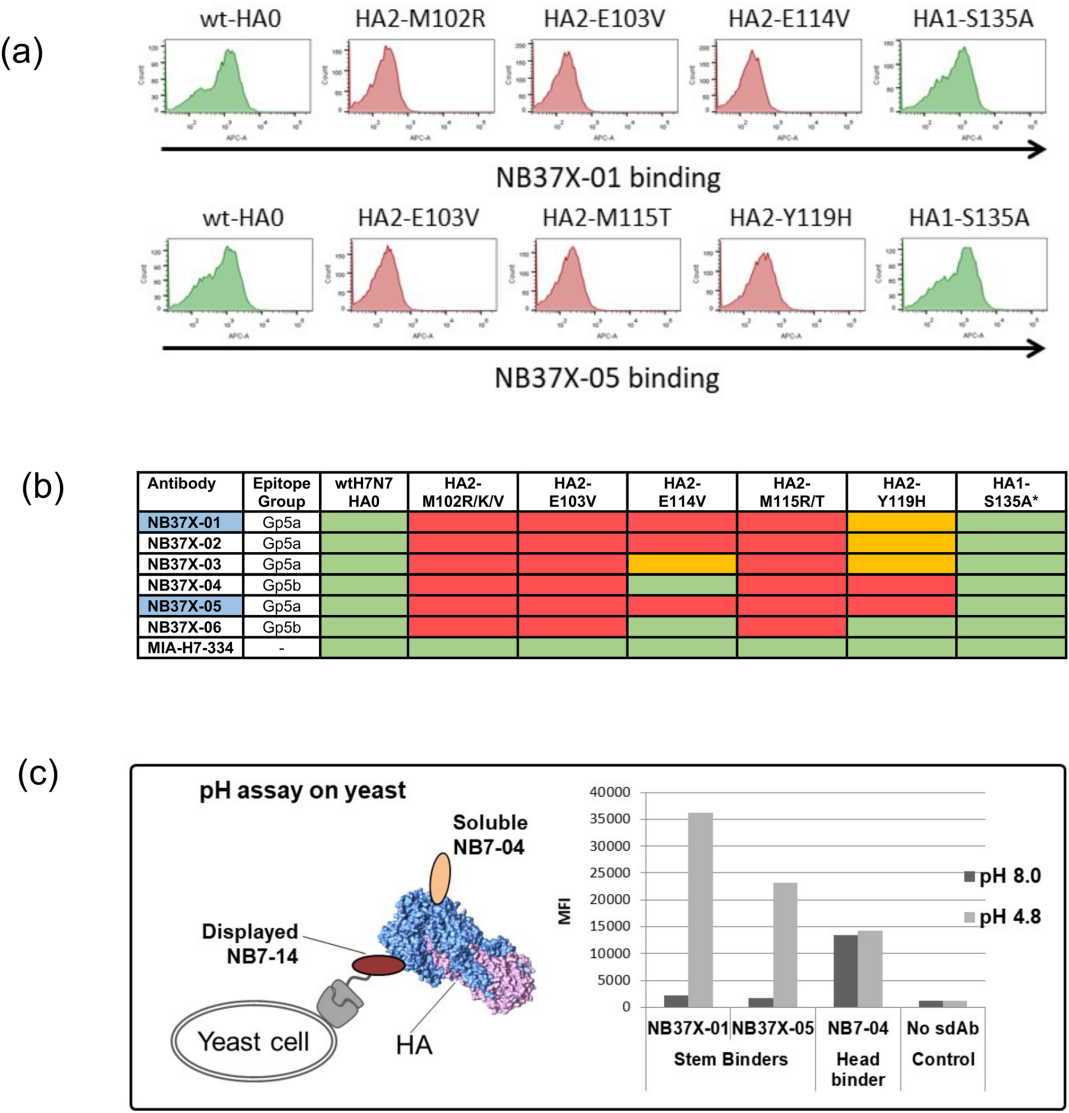
Epitope footprint of H3/H7 cross-reactive nanobodies. (**a**) Flow cytometry histograms (FlowJo 10.4 software) showing NB37X-01 and NB37X-05 binding to displayed wild type H7-HA0 and mutants selected (**b**) Nb binding activity to a panel of yeast displayed HA0 mutants. Residue numbering is relative to HA2 domain. The Nbs used for epitope mapping are highlighted in blue. Commercial antibody MIA-H7-334 is included as positive control and retains binding to all mutants. Relative binding of Nbs to each displayed mutant were categorized as follows: ≤ 15% no binding (red), between 15 and 40% intermediate binding (orange) and ≥ 40% strong binding (green). *Represents retention of binding to the HA1 head domain carrying a non-relevant mutation HA1-S135A (**c**) yeast cells displaying the head specific Nb NB7-14 were incubated with HA treated with low and neutral pH and binding of the stem specific Nbs NB37X-01 and NB37X-05 was detected as mean fluorescent intensity (MFI). NB7-04 was a non-competing head specific nanobody control.

**Figure 5:**
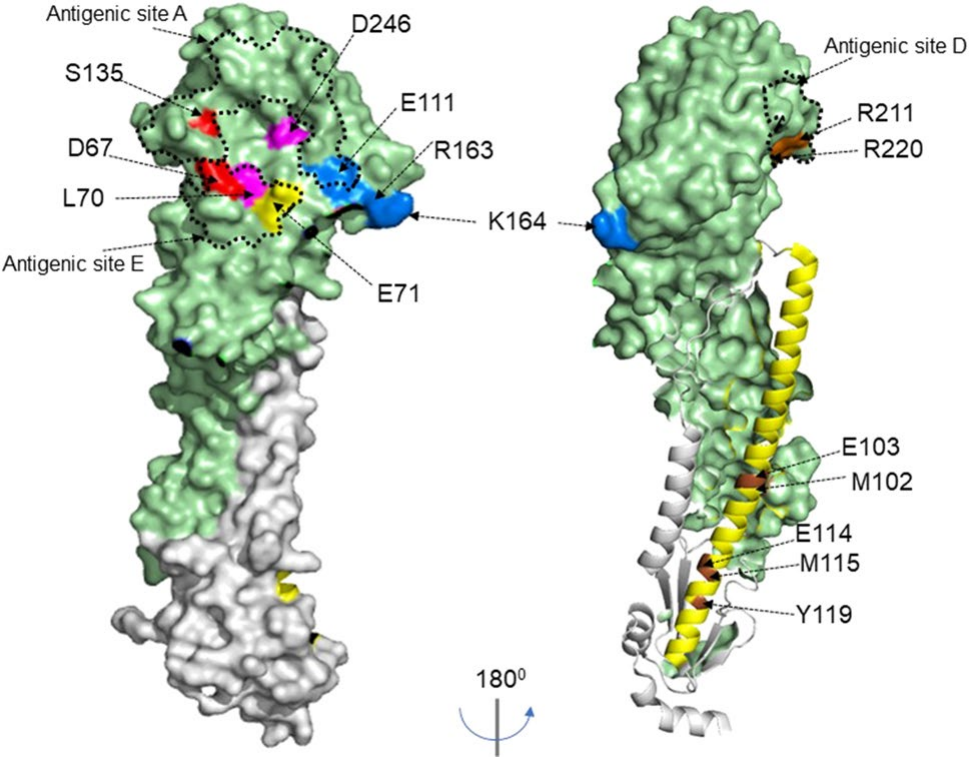
HA structure showing nanobody epitope groups. Surface structure of hemagglutinin H7-HA [A(H7N9) A/Shanghai/02/2013, (PDB file 4LN6)] showing key residues for each epitope group using Pymol 2.3.1 software. The structure shows HA1 domain (light green), HA2 domain (grey), and key residues for each epitope group, Gp1a (red), Gp1b/1c/1d (pink), Gp2 (yellow), Gp3 (blue) and Gp4 (brown). HA2 helix is shown in yellow with Gp5 epitopes residues highlighted in brown on the helix and numbering for the HA stem starts at sequence ‘GLFG…’.Previously described antigenic sites A, C and D ^47^ on group 2 HA are indicated with dotted lines.

The cross-reactive Nbs NB37X-01 and NB37X-05 were shown to bind only full length H7-HA0 with the absence of binding to the H3-HA1 head domain (Fig. S3) suggesting the epitope(s) lie(s) within the more conserved stem region. The H7-HA0 library was selected separately with NB37X-01 and -05, and identified five residues (M102, E103, E114, M115, Y119) that specifically interfered with Nb binding (Fig. 4a,b) which all mapped to the HA stem. None of the HA stem mutations had any effect on the binding of the Nbs (NB7-01 to 17) which have epitopes in the head domain. In addition, both M102 and M115 were mutated to more than one residue reflecting the importance of these residues. The entire panel of cross-reactive Nbs showed loss of binding to all 5 mutants with the exceptions of NB37X-04 and NB37X-06, which retained binding to the HA2-E114V mutation. This suggests they have a common physical footprint within the stem domain (Fig. 5). However, they could be placed in two epitope subgroups (Gp5a and Gp5b) based on the differential effect of the HA2-E114V mutation. This cross-reactive epitope sits within the HA2 helix of the stem which has a crucial role in influenza infection by mediating a large conformational change in the acidified endosomal compartment to initiate membrane fusion. We evaluated if binding of the heterosubtypic H3/H7 cross-reactive stem binding Nbs could be affected by this conformational change by mimicking it in vitro through exposure of HA to low pH. We used a yeast display based sandwich assay where we sub-cloned the H7-specific antibody NB7-14 into the yeast display vector to bind HA via the head domain and treated HA with different pH buffers followed by probing with the putative stem binding Nbs (Fig. 4c). All cross-reactive Nbs (NB37X-01 to 06) showed a substantial increase in binding upon low-pH treatments whereas the head binder NB7-04 bound HA at both pH 4.8 and pH 8.0 equally well (Fig. 4c). As NB7-04 and NB7-14 share an overlapping epitope footprint in the head domain the binding seen with the head specific nanobody indicates that trimeric HA is being detected in this assay format. This confirms that the cross-reactive Nbs bind to the HA stem region and suggests that they have preferential binding to the low pH conformation.

### Correlation of nanobodies belong to epitope Gp3 to natural sequence divergence

**Figure 6:**
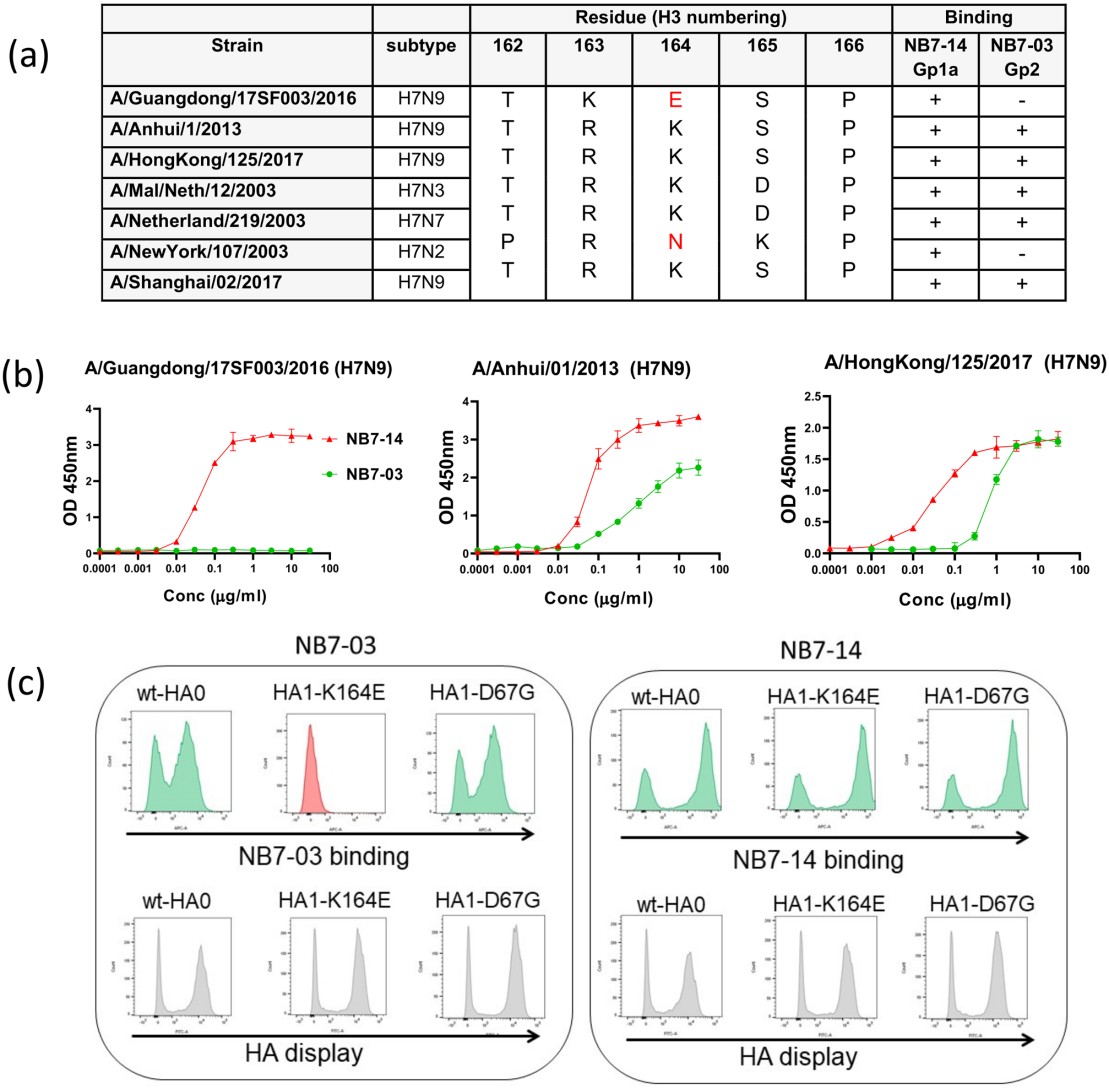
Correlation of epitope Gp2 specificity to natural sequence divergence. (**a**) Correlation of binding of NB7-03 (Gp3) and NB7-14 (Gp1a) to natural sequence divergence at K164. (**b**) ELISA of purified NB7-14 (red) and NB7-03 (green) binding to inactivated whole virus antigen preparations from the following: A/Guangdong/17SF003/2016 (H7N9); A/Anhui/01/2013 (H7N9) and A/Hong Kong/125/2017 (H7N9). (**c**) Confirmation of specificity of NB7-03 and NB7-14 to yeast cells displaying the HA1-K164E mutation.

Epitope group Gp3, comprising NB7-03, NB7-10 and NB7-13, were unique as these Nbs did not show any binding to the most recent highly pathogenic avian influenza A(H7N9) strain A/Guangdong/17SF003/2016 and the human H7N2 strain (A/NewYork/107/2003). The epitope footprint of these Nbs comprises E111, R163 and K164, and sequence comparison of the strains tested in ELISA identified this as a region of natural sequence divergence (Fig. 6) These two influenza strains have a naturally occurring substitution at K164 to either a glutamate (E) or asparagine (N) (Fig. 6). Of particular interest was our being able to select the naturally occurring HA1-K164E substitution from a yeast displayed error prone PCR HA library. This artificially generated mutation correlated with a naturally occurring sequence difference between the prototype low pathogenic and high pathogenic strains of A(H7N9)^40^. This suggests that yeast display may be used as a tool to investigate the evolution of the HA molecule under different selective pressures without the need to use live virus and high containment facilities.

## Discussion

To date there have been 5 epidemic waves of zoonotic infections of influenza A(H7N9) with up to 1568 reported cases of human infection (https://www.who.int/influenza/vaccines/virus/201909_zoonotic_vaccinevirusupdate.pdf?ua=1) with a 40% fatality rate. During the 5th wave in 2016–17, the emergence of highly pathogenic avian influenza (HP) A(H7N9) raised significant concerns as unlike (LP) A(H7N9) it maintained the capacity to infect via both human and avian receptors^41, 42^. In preparation for any further adaptations in H7N9 which could threaten a pandemic, vaccines, anti-viral drugs and monoclonal antibodies which can be used for control, surveillance and as emergency prophylactics are essential.

In this study we have isolated a panel of homo-subtypic H7 and hetero-subtypic H3/H7 cross-reactive Nbs. We demonstrate that alpacas immunised with a single H7 strain can yield Nbs with broad reactivity which can maintain binding and resistance to natural antigenic changes over a minimum of 17 years. We have also isolated Nbs which can distinguish between the most recent high pathogenicity avian influenza strain (HP) A/Guangdong/17SF003/2016 and the low pathogenicity (LP) A/Hong King/125/2017 (Fig. 6). In many cases Nbs were very high affinity (less than 1 nM) reflecting extensive somatic hypermutation and optimisation by the alpaca immune system. This could be expected as to date alpaca’s are not thought to be a reservoir for IAV so should not have any prior immune history of exposure to group 2 viruses^32^. This is in contrast to many of the human monoclonal antibodies to H7N9 where very few somatic mutations are seen, suggesting the antibodies are derived from an early, essentially germline response, to the virus^7, 19^.

To understand the functional significance of Nb cross-reactivity we have mapped their epitopes using yeast display and mutational scanning. This approach is not limited by the need to maintain infection as is the case with conventional viral escape assays which uses live influenza virus and high containment facilities^28^. Using yeast display we are able to do a comprehensive mutational scan which encompasses both physical and functional epitope footprints, which are only limited by the need to preserve a folded, conformationally correct HA molecule anchored to the yeast cell surface^28^. Our sequential analysis of selection on individual Nbs to identify HA mutations which disrupt binding, followed by screening on the wider Nb panel, has allowed the Nbs to be placed into 5 major epitope groups (Gp1, Gp2, Gp3, Gp4 and Gp5). Epitope group Gp1 was the largest group with 11 different Nbs which could be further divided into 4 sub-groups which, although sharing D67 as a key epitope residue (Fig. 5), showed clear distinctions in their physical footprint. This is consistent with each Nb having unique paratopes determined principally by their VHH-CDR3 sequence and being derived from different ancestor B cells. It is interesting to speculate that this information may be used to predict different trajectories for HA escape from binding and be useful in choosing optimum combinations of antibodies. This is an important consideration in designing multi-domain antibodies or antibody cocktails for therapeutic applications where targeting multiple epitopes has the potential to reduce the risks of viral escape. The generation of multi-domain antibodies using conventional monoclonal antibodies is complex, principally due to the antigen combining site requiring the stable pairing of two separate light chain and heavy chain polypeptides, thus chain-switching and product heterogeneity can be a problem in their production. In contrast, one of the advantages of Nbs is that all antigen activity is focussed on one independently folding binding unit which can be easily linked with other such binding units^29, 33, 37^. We have shown that by simple linking together of two NB7-14 binding units we can enhance its potency over 1000-fold to and IC50 of 113 pM (Fig. 2). This also highlights a simple route to multi-paratopic nanobodies using Nbs belonging to different epitope groups which could be compared for mutational escape using yeast display^37^. Multi-specific nanobodies to different epitopes on H7 could be stockpiled and bridge the gap until a vaccine is available during a pandemic emergency. Nanobodies have shown themselves to be capable of potent neutralisation of a range of different viruses including HIV-1^43^, RSV^44^, rabies^45^ and influenza virus^29, 38^. Broad neutralising Nbs to pandemic influenza viruses could be expected to have considerable potential for therapy or prophylaxis and we have recently shown adeno-associated viral vector delivery is able to drive sustained high level expression in situ and protection of mice from both pandemic H1N1 and highly pathogenic avian influenza H5N1^46^.

Human seasonal influenza A(H3N2) has been previously characterised as having 5 distinct antigenic sites (A, B, C, D, and E)^47^. The nanobody epitope Gp1 overlaps with antigenic sites E and A whereas epitope Gp4 overlaps with antigenic site D (Fig. S5). In analysing the diversity of 324 H7N9 sequences (Table S1), we saw a high degree of conservation in the epitope footprint of Gp1 Nbs with D67, L70 and S135 being absolutely conserved. The epitope group Gp1 is adjacent to the receptor binding site of HA and overlaps with antigenic site A, which is highly conserved amongst many H7 viruses, including strains of both Eurasian and North American lineages^48, 49^. Several human monoclonal antibodies which bind in this region from human donors exposed to H7N9 A/Shanghai/02/2013 and A/Anhui/01/2013 vaccines^7, 19^ have been shown to provide protection against H7N9 challenge, demonstrating the functional importance of this epitope^48, 50^ and their potential as therapeutics. Nbs, such as NB7-14, which map to this region (Fig. S5) may also similarly have potential as a therapeutic or emergency prophylactic.

Nbs with H3/H7 cross-subtype reactivity and enhanced binding at low pH were located to the highly conserved HA stem and belonged to epitope Gp5 (Fig. 4) (Table S1). Previous studies have described 66.5% sequence identity between H3 and H7 subtypes in the HA2 domain compared to 37% in the HA1 head domain^51^. The stem region is crucial in mediating viral infection and undergoes a large structural re-arrangement in the acidified environment of the endosomal compartment. It has been proposed that H106 and adjacent residues comprising M102 and E103 within the Gp5 epitope footprint function as a sensor for the enhanced protonation of HA at low pH^15^. The increase in binding we see at low pH is consistent with Nbs showing preferential binding to the HA stem after it has gone through the specific pH dependent conformational change. Non-neutralising human antibodies with a pH sensitive binding have been described which correlated with increased phagocytosis in vivo^20^. As Nbs NB37X-01 to 06 bind to a similar pH sensitive epitope they may be capable of similar mechanisms of viral neutralisation after appropriate reformatting as Fc-fusions to mediate interactions with phagocytic effector cells^46, 52, 53^.

Nanobodies binding to epitope group 3 had a unique specificity which doesn’t overlap with any of the previously described antigenic sites from human immune responses to H7N9 influenza^47^ and may reflect a uniqueness to the immune response in alpacas that has not been shaped by prior exposure to IAV. This binding specificity could be correlated to natural sequence divergence at K164 between low (LP) and high pathogenicity (HP) H7N9 influenza viruses. This is a further example of how yeast display and mutational scanning is able to correlate Nb specificity with natural sequence change, which, if applied predictively, could be used to assess the suitability of stockpiled nanobody reagents as new viruses emerge^28, 30^. To our knowledge we have described the first Nbs capable of distinguishing (HP) A/Guangdong/17SF003/2016 (H7N9) strain from (LP) A/Hong Kong/125/2017 (H7N9) and have been able to correlate this specificity to natural sequence divergence at K164 in the HA1 head domain. Since H7N9 presents in poultry with only mild symptoms, early warning signs of a pandemic may be extremely difficult to detect and the availability of specific Nbs formatted for rapid detection of this antigenic difference may be useful for influenza surveillance in the field using low cost biosensors^54^.

In summary, we have isolated a panel of nanobodies to A(H7N9) which have been grouped on the basis of their epitopes and correlated this with their specificity and functional activity. They may be useful in monitoring the antigenic changes that occur in the HA of A(H7N9) during transmission in poultry, controlling the spread of virus in birds, or as emergency therapeutics in humans.

## Methods

### Reagents and influenza antigens

Whole influenza virus inactivated antigen standards (National Institute for Biological Standards and Control, NIBSC) used in this study were derived from strains: A/New York/107/2003 NIBRG-109 (H7N2) (08/362), A/Anhui/01/2013 NIBRG-268 (H7N9) (14/250), A/Mallard/Netherlands/12/2000 NIBRG-60 (H7N3) (07/336), A/Uruguay/716/2007 NYMC X-175C (H3N2) (08/278), A/Guangdong/17SF003/2016 NIBRG-375 (H7N9) (18/196), A/Texas/50/2012 (H3N2) (13/116) and A/HongKong/125/2017 (H7N9) (IDVDV-RG58B, CBER-reference antigen #88),. Purified recombinant hemagglutinins were full length HA0: A/Brisbane/10/2007 (H3N2), A/chicken/Netherlands/1/2003 (H7N7), A/Netherlands/219/2003 (H7N7), A/Shanghai/1/2013 (H7N9) (Protein Sciences) and HA1 (aa 26-344) A/chicken/Netherlands/1/2003 (H7N7), A/Hong Kong/125/2017 (H7N9) (e-ENZYME). Reverse genetic influenza strain A/Anhui/01/2013 (H7N9) NIBRG-268 was used.

### Construction and selection of phage and yeast displayed libraries

All methods performed on animals were carried out in accordance with relevant guidelines and regulations. All experimental protocols were reviewed and approved by the National Institute for Biological Standards (NIBSC) ethics committee and performed under United Kingdom Home Office License (PBF57622). A juvenile male alpaca was purchased through the Royal Veterinary College, Hertfordshire, UK. Primary immunisation and each subsequent boost was with 50 µg of purified H7-HA from A/Netherlands /219/2003 (Protein Sciences) and sample collection was performed as in^29^. For antibody library construction approximately 10 ml samples of blood were collected from a immunised alpaca into heparinised tubes. Peripheral blood lymphocytes were purified using a ficol hypaque centrifugation procedure (Sigma-Aldrich) and RNA was extracted using a RiboPure RNA extraction kit (Novagen) according to manufacturer’s instructions. First strand cDNA synthesis was performed using Superscript III reverse transcriptase (Invitrogen) and oligo-dT primer with 200 ng of total RNA per reaction. A phage displayed library was constructed in pNIBS-1 vector as described previously^29^. This vector allows both phage display and production of soluble VHH nanobody fragments appended with a Histidine purification tag and a c-Myc epitope tag for detection of binding in ELISA.

Phage antibody library selections were performed essentially as in^29^ using immunotubes (Thermo Fisher Scientific) coated overnight at 4 °C with 1 ml of 10 µg/ml recombinant H7-HA (Protein Sciences) or whole influenza virus inactivated antigen standards (NIBSC) reconstituted in PBS. Bound phage antibodies were eluted by adding 1 ml of 100 mM triethylamine followed by incubation for 10 min on a rotating platform at room temperature. The eluted phage were neutralised with 0.5 ml 1 M Tris-HCl pH 7.5. To amplify the selected phage for subsequent rounds of selection 1 ml of eluted phage were mixed with 5 ml of an *Escherichia coli* ER2738 (Agilent Technologies) culture grown to an OD_600_ of 0.5 and 4 ml of 2 × TY media followed by incubation in a water bath at 37 °C for 30 min. This was then spread onto 22 cm bioassay dishes containing 2 × TY agar supplemented with 100 µg/ml (w/v) carbenicillin and 2% (w/v) glucose. Plates were grown overnight at 37 °C and harvested and the phage titres before and after selection were determined^29^.

Yeast antibody libraries were generated using the same cDNA used for the construction of the phage libraries as template. Antibody genes were amplified with oligonucleotides: Alp_FR1_*Sfi*1_YRec (gggcggaggatctggtggcggaggttctgcggcccagccggccATGGCACAGKTGCAGCTCGTGGAGTCNGGNGG) and Alp_FR4back_*Not*1_YRec (tccaaacccaacaatgggtttgggattggcttaccagcggccgc TGAGGAGACGGTGACCTG), where uppercase sequences anneal with antibody sequence extended lowercase sequences are included for subsequent yeast recombination and *Sfi*1/*Not*1 restriction sites are underlined. For each library, 20 µg of amplified PCR product was co-transformed with 20 µg of *Sfi*1/*Not*1 digested pNIBS-5 vector^28^ into *S. cerevisiae* EBY100 competent cells. pNIBS-5 is a yeast display vector which allows the display on yeast of proteins appended with a N-terminal SV5 epitope tag for detection. The final library size was determined through serial dilutions on selective plates as 2.25 × 10^7^ clones. Standard procedures and recipes for growth, induction, yeast cell labelling, media and buffer preparation were used^28^. For yeast cell analysis and sorting we used 70 nM recombinant HA (eENZYME) followed by primary reagents monoclonal mouse anti-SV5 (Bio-Rad, MCA1360) and biotinylated monoclonal rabbit anti-His (Bethyl Laboratories, A190-114B) and by secondary reagents monoclonal goat anti-mouse IgG AlexaFluor488 (Thermo Fisher, Scientific, A11029) and streptavidin AlexaFluor647 (Thermo Fisher Scientific, S32357). For sorting ~ 10^8^ cells were labelled in the first round and 50,000 events were sorted decreasing to 5,000 events for subsequent rounds. Yeast plasmid DNA was purified from the final outputs and transformed into TG1 electro-competent *Escherichia coli* cells and 24 single bacterial colonies were sequenced to identify there VHH-CDR3 sequence. All yeast cell analysis and sorting was performed on BD Canto II or BDAria III flow cytometers (Becton Dickinson) respectively. Data was analysed using FlowJo 10.4 software^28^.

### Nanobody expression and screening

Primary screening was carried out in a 96 well ELISA plate (Nunc) coated with recombinant HA at 1 µg/ml overnight in PBS at 4 °C^29^. Influenza virus antigen standards (National Institute for Biological Standards and Control, NIBSC) were reconstituted in 1 ml sterile water or PBS and then diluted 1/200 in 0.5 M bicarbonate buffer pH 9.6 prior to incubation overnight at 4 °C in a 96 well plate (Thermo Fisher Scientific). For primary screening soluble VHH antibodies were harvested from culture supernatants in a 96 well format. In short individual colonies from each round of selection were inoculated into 100 µl of 2 × TY medium supplemented with 100 µg/ml (w/v) carbenicillin and 2% (w/v) glucose in a 96 well flat bottom plate (Corning) using sterile toothpick and grown overnight in a shaking incubator at 30 °C. From this master plate a new 96 well round bottom plate containing 100 µg/ml (w/v) carbenicillin and 0.1% (w/v) glucose was grown at 37 °C for 6 h until OD 600 of approximately 0.9 was reached after which time 30 µl of 2 × TY supplemented with 100 µg /ml (w/v) carbenicillin plus 5 mM IPTG (1 mM final concentration) was added and incubation continued overnight. The plates were then centrifuged at 600 × g for two minutes and supernatants containing the soluble VHH nanobodies were harvested and tested in ELISA. After addition of 100 µl of nanobody to individual wells and incubation for 1.5 h at room temperature, wells were washed with PBS and PBS-tween 0.1% (v/v) before 100 µl of anti c-myc 9E10-HRP (1/1000 dilution) (Roche) in 2% (w/v) milk powder was added and binding detected at OD 450 nM using TMB after 15 min incubation at room temperature. In all ELISA’s a negative control nanobody with no known specificity was used. For their large-scale production, cloned VHH gene fragments were transformed into the non-suppressor *Escherichia coli* strain WK6, expressed and purified^28, 29^. Nanobodies were purified from periplasmic preparations using immobilised metal chelate chromatography from the periplasmic fraction using Talon resin (Clontech). Eluted antibodies were dialysed against PBS using dialysis cassettes of molecular weight cut-off of 3 kDa (Thermo Fisher Scientific)^29^.

Nbs were converted into bivalent molecules using a (G4S)6 linker to fuse two Nb binding domains ‘head to tail’. Sequences of each nanobody unit were optimised to limit the percentage of GC content and to reduce internal homology within the construct. Constructs were assembled by PCR from overlapping oligonucleotides, sub-cloned into pNIBS-1 and transformed into the *Escherichia coli* strain WK6. Bivalent nanobodies were expressed and purified as above.

### Lentiviral pseudotype assays

Lentiviral pseudotypes were produced by transient co-transfection of HEK293T/17 cells using polyethylenimine. Plasmid p8.91 encodes the structural (gag, pol) genes and pCSFLW represents the genome incorporated into the pseudotypes bearing the firefly luciferase reporter. Influenza A hemagglutinin genes, in the expression plasmid pI.18 were also added to this mix alongside the Human Airway Trypsin (HAT) expression plasmid, pCAGGS-HAT, to allow for HA0 to HA1/2 maturation. The pseudotype based micro-neutralisation assay (pMN) was carried out in Nunc F96 microplates (Thermo Fisher Scientific). 1:2 serial dilutions of nanobodies were performed across the 96-well plate in a total of 50 µl DMEM + 10% (w/v) fetal bovine serum and 1% (w/v) penicillin/streptomycin. HIV-1 derived lentiviral pseudotypes bearing influenza HA were then added to yield a relative luminescence unit (RLU) input of 1.5 × 10^6^ per well, in a total volume of 50 µl. Plates were then incubated in a humidified incubator at 37 °C, 5% CO_2_ for one hour, after which 1.5 × 10^4^ HEK293T/17 cells were added per well in a total volume of 50 µl. After 48 h, supernatants were removed and a 50:50 mix of PBS and Bright-Glo (Promega Corporation) was added to each well. Plates were incubated at room temperature for five minutes and then luminescence was read using a Glomax luminometer (Promega Corporation). Signals were normalised to cell and virus only controls, representing 100% and 0% neutralisation respectively. IC_50_ values were calculated by non-linear regression using GraphPad Prism.

### Analysis using surface plasmon resonance

For binding and affinity ranking against different full length recombinant HA0 and HA1 head domains we used single cycle kinetics on a Biacore T100 machine (GE Healthcare)^39^. In brief, HA was immobilised onto a Biacore CM5 chip in 10 mM sodium acetate pH 5.5 using an amine coupling kit (GE Healthcare) to create surface densities of between 1000 and 3000 RU. A concentration series of purified nanobody were sequentially run over the different antigen surfaces ranging from 1 to 25 nM. A reference surface was subtracted prior to evaluation of the sensograms using the single cycle kinetics procedure of the Biacore T200 evaluation 3.1 software (GE Healthcare) in combination with a 1:1 fitting model.

### Yeast display of hemagglutinin, error-prone library construction and screening

Full length HA0, HA1 and HA2 (A/Netherlands/219/2003 (H7N7, D1-V508 mature protein numbering, HA1: D1-R323, HA2 G1-V185) respectively, were codon optimized for yeast expression and synthesised, including *Sfi*I-*Not*I restriction sites (Integrated DNA Technologies), cloned into the yeast display vector pNIBS-5 and transformed into *S. cerevisiae* EBY100^28^. Standard procedures and recipes for growth, induction, yeast cell labelling, media and buffer preparation were used. Staining with purified Nbs was performed by incubating cells with 100 nM of Nbs followed by monoclonal mouse anti-SV5 (Bio-Rad, MCA1360) and polyclonal chicken anti-cMyc (Bethyl Laboratories, A190-203A) followed by secondary reagents monoclonal goat anti-mouse IgG AlexaFluor488 (Thermo Fisher Scientific, A11029) and goat anti-chicken IgG AlexaFluor647 (Jackson ImmunoResearch, 103-605-155). Anti-influenza control H3 and H7 specific antibodies MIA-H7-334 and MIA-H3-501 were used (eEnzyme). Labelled yeast cells were analysed on BD Canto II or BDAria III flow cytometers.

Error prone PCR amplification of the HA gene was carried out using a GeneMorph mutagenesis kit according to manufacturer’s instruction (Agilent Technologies) and 20 µg of mutated PCR product was co-transformed with 20 µg of *Sfi*1/*Not*1 digested pNIBS-5 yeast display vector^28^ into *S. cerevisiae* EBY100 competent cells (Invitrogen). The final library size was determined through serial dilutions on selective plates, and yeast plasmid minipreps of the library was transformed into TG1 electro-competent *Escherichia coli* cells (Agilent Technologies) for single colony sequencing. Individual colonies were picked and sequenced to assess mutation frequency. The yeast library was grown in selective medium for induction of HA display. 10^8^ cells were co-stained with 100 nM Nb, followed by anti-SV5/anti-cMyc antibodies to detect Nb binding and HA display using fluorescent secondary reagents as described above. Flow cytometric cell sorting was performed using BDAria III. For the first round of a sorting a gate was chosen to sort cells displaying HA (by virtue of anti-SV5 signal) but absence of nanobody binding (lower right quadrant of a FACS dot plot). A second round was performed using the same sorting conditions. A third round of ‘positive’ sorting was done using 200 nM of a non-competing nanobody of NB37X-01 for epitope mapping of HA1 specific nanobodies (NB7-14, NB7-08, NB7-15, NB7-03, NB7-05) and NB7-14 for epitope mapping of the stem specific nanobodies (NB37X-01, NB37X-05). Yeast plasmid DNA was purified from the final outputs and transformed into TG1 electro-competent *Escherichia coli* cells and single bacterial colonies were sequenced. Sequence reads were assembled and aligned to the wild-type HA0 or HA1 genes to identify candidate mutations. Yeast clones with defined mutations were separately labelled for HA display and Nb binding. The Nb binding mean fluorescence intensity (MFI) of each Nb-mutant HA pair was divided by the MFI value of the wild-type H7-HA interaction, and the resulting ratio normalised to percentage values. Binding was categorized as follows, ≤ 15% no binding, between 15 and 40% intermediate binding and ≥ 40% strong binding (green).

## Supplementary Information

Supplementary Information.

Supplementary Figure S5.
