# Supplementary material for: Nanobodies mapped to cross-reactive and divergent epitopes on A(H7N9) influenza hemagglutinin using yeast display
Source: Sci Rep. 2021 Feb 4;11:3126. doi: 10.1038/s41598-021-82356-4 (PMC7862619; doi:10.1038/s41598-021-82356-4)
Supplement: Supplementary file 1 — Supplementary Information. [file 41598_2021_82356_MOESM1_ESM.pdf]

Supplementary Information

Nanobodies mapped to cross-reactive and divergent epitopes on A(H7N9) influenza hemagglutinin using yeast display.

[1]Tiziano Gaiotto, [1]Walter Ramage, [1]Christina Ball, [1]Paul Risley, [3]George W Carnell, [3]Nigel Temperton, [2]Othmar G Engelhardt, [1]Simon E Hufton*

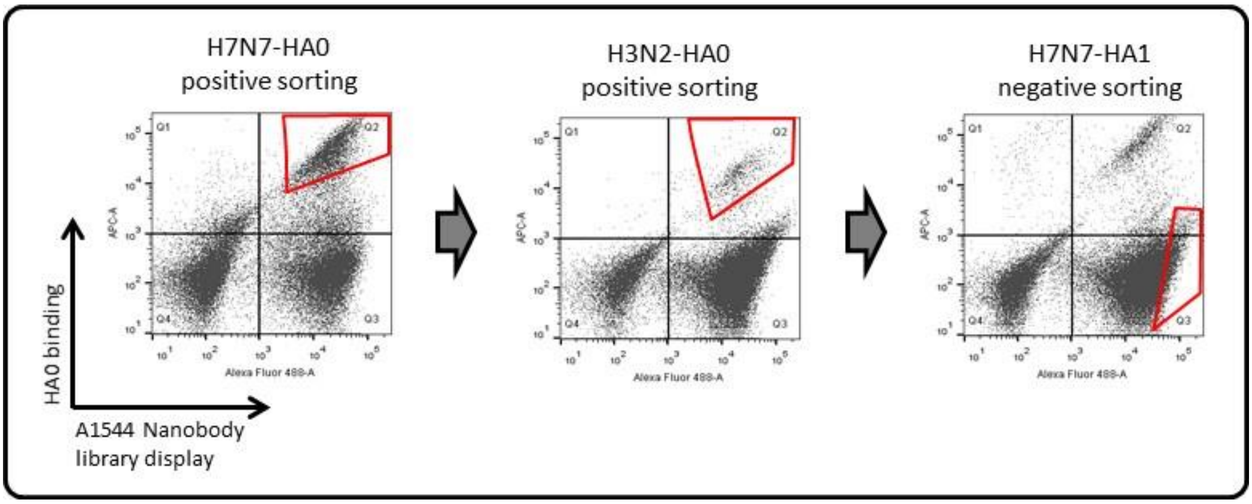

Fig S1. Guided yeast library cell sorting for stem specific binding nanobodies.
FACS plots (FlowJo 10.4 software) showing the initial positive sorting of yeast Nb library using recombinant H7N7 HA0 (A/chicken/Netherlands/1/2003), followed by a second round of positive sorting using recombinant H3N2 HA0 (A/Brisbane/10/2007) and by a third round of negative sorting using recombinant H7N7 HA1 (A/chicken/Netherlands/1/2003). For each round the gated population for cell sorting is shown in red.

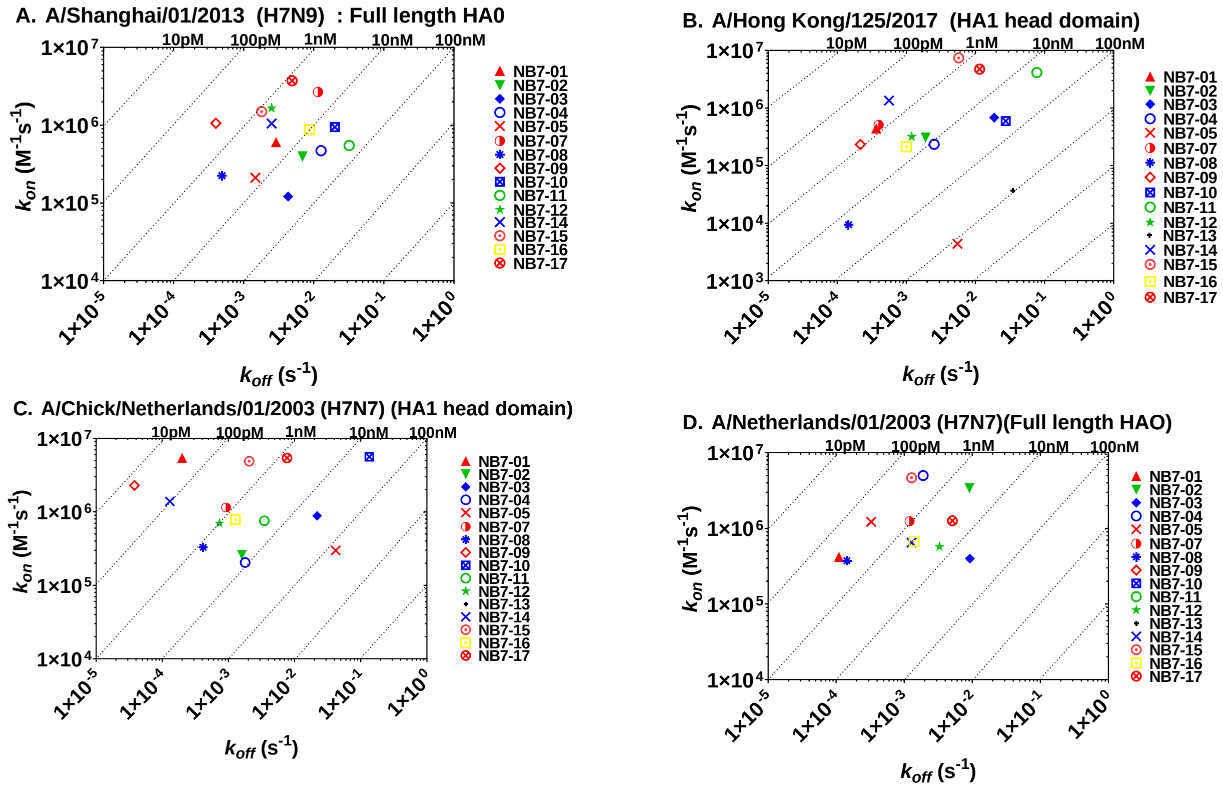

Fig S2. Binding constants (*k_{on} and k_{off}*) on recombinant HA0 and HA1. Single cycle kinetic data are presented as rate plots with iso-affinity diagonals (RAPID) where the diagonals (dotted lines) are connecting the points of equal dissociation constant (K_D). Affinity on (A) recombinant HA0 from A/Shanghai/01/2013 (H7N9), (B) recombinant HA1 head domain from A/Hong Kong/125/2017 (H7N9) (C) recombinant HA1 head domain from A/Chick/Netherlands/01/2003 (H7N7)(D) recombinant HA0 from A/Netherlands/219/2003 (H7N7). Fitting was with a 1:1 Langmuir fitting model using Biacore T200 evaluation software 3.1 software. Equilibrium dissociation constants (K_D) are given in Table 2.

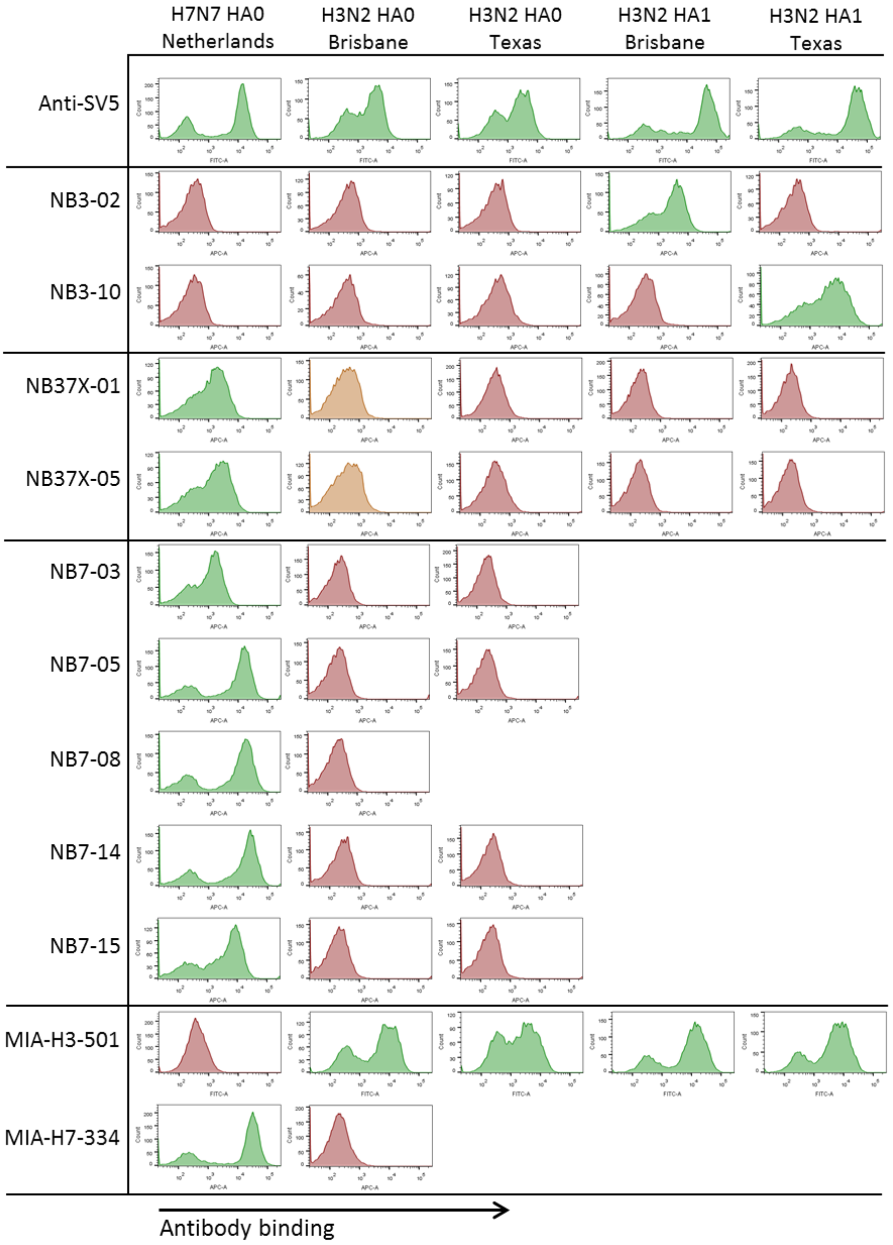

Fig S3. Nb binding to yeast displayed wild type HA (full-length HA0 or head domain HA1). Anti SV5 measures HA display via epitope tag. NB3-02 and NB3-10 are nanobodies against H3-HA1 domain as controls to confirm display of H3N2-HA1 on yeast. NB37X-01 and NB37X-05 are cross reactive nanobodies. NB7-03, NB7-05, NB7-08, NB7-14, NB7-15 are nanobodies specific to H7-HA. MIA-H3-501 is control mAb specific for H3N2-HA0. MIA-H7-334 is control mAb specific for H7N7-HA0. HA0 precursor gene of A/Brisbane/10/2007 (H3N2) (Q1-D487 mature protein numbering). HA1 head domain of A/Brisbane/10/2007 (H3N2) (Q1-R329). HA0 precursor gene of A/Netherlands/219/2003 (H7N7) (D1-V508 mature protein numbering). HA0 precursor gene of A/Texas/50/2012 (H3N2) (Q1-D512 mature protein numbering). HA1 head domain of A/Texas/50/2012 (H3N2) (Q1-R328). Green histograms indicate binding, red is no binding and amber is partial binding.

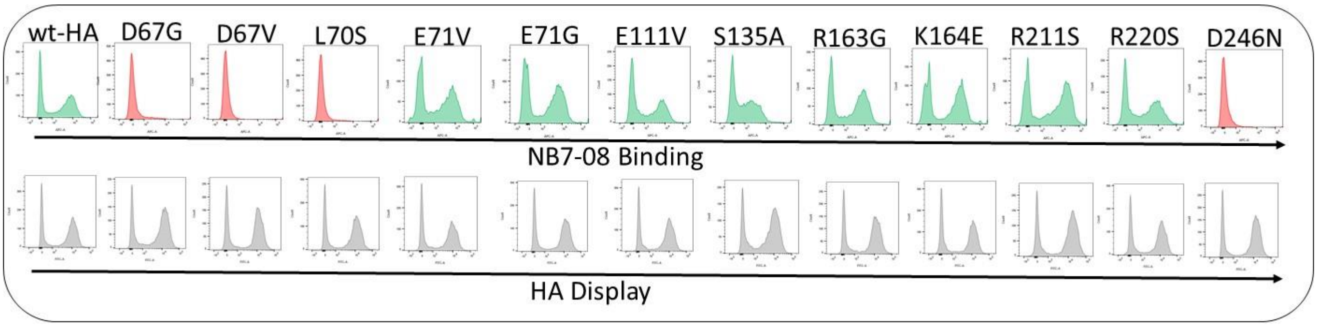

FIG S4. Example flow cytometry histograms showing NB7-08 binding to yeast displayed wild-type HA and complete panel of HA mutants. Green indicates mutation has no effect on NB7-08 binding, red indicate NB7-08 binding is lost. Grey histograms indicate HA display and show mutations do not affect the level of HA display.

Table S1. H7N9 amino acid variability at the 5 distinct nanobody binding epitopes.

Ag site*	E			A	-	A	-		D	-	Stem				
	Gp1a/1b/1c/1d/ Gp2					Gp3			Gp4		Gp5				
aa**	D67	L70	E71	S135	D246	E111	R163	K164	R211	R220	M102	E103	E114	M115	Y119
A															
C															1
D	324				312			4							
E			313		11	324		3[a]				319	324		
F															
G															
H															
I										7					
K			11				2	286							
L		324													
M											323			324	
N								26[b]							
P							3								
Q															
R							317		324	317					
S				324				7							
T							2				1				
V					1							5			
W															
Y															323
X															

* Antigenic site A/B/C/D/E (H3 numbering) [1]

**H7 amino acids numbering is from the first residue of the mature protein with DKIC

A total of 324 complete protein sequences corresponding to H7N9 strains were retrieved from NCBI influenza database.

a. K164E substitution present in A/Guangdong/17SF003/2017 (H7N9).

b. K164N substitution present in A/NewYork/107/2003 (H7N2)

1. Yang, H., et al., *Structural and Molecular Characterization of the Hemagglutinin from the Fifth-Epidemic-Wave A(H7N9) Influenza Viruses.* J Virol, 2018. **92**(16).

2. Wiley, D.C., I.A. Wilson, and J.J. Skehel, *Structural identification of the antibody binding sites of Hong Kong in influenza haemagglutinin and their involvment in antigenic variation*. Nature, 1981. **289**(5796): p.373-8
