# Supplementary figures and images for: Nanobodies mapped to cross-reactive and divergent epitopes on A(H7N9) influenza hemagglutinin using yeast display
Source: Sci Rep. 2021 Feb 4;11:3126. doi: 10.1038/s41598-021-82356-4 (PMC7862619; doi:10.1038/s41598-021-82356-4)

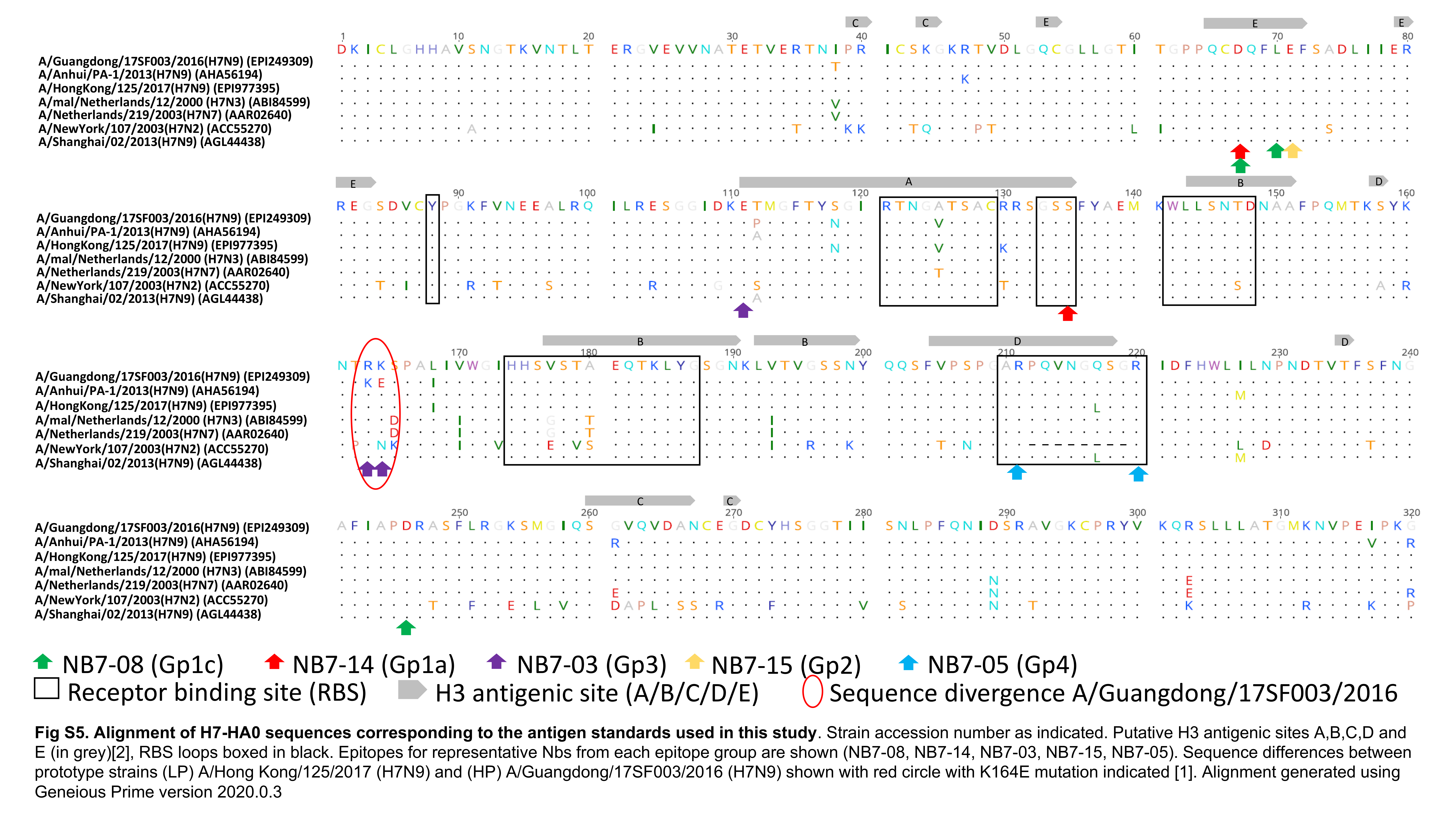

Supplement: Supplementary file 2 — Supplementary Figure S5. [file 41598_2021_82356_MOESM2_ESM.jpg]
